# Over-Expression of a Rice Tau Class Glutathione S-Transferase Gene Improves Tolerance to Salinity and Oxidative Stresses in Arabidopsis

**DOI:** 10.1371/journal.pone.0092900

**Published:** 2014-03-24

**Authors:** Raghvendra Sharma, Annapurna Sahoo, Ragunathan Devendran, Mukesh Jain

**Affiliations:** Functional and Applied Genomics Laboratory, National Institute of Plant Genome Research (NIPGR), Aruna Asaf Ali Marg, New Delhi, India; National Taiwan University, Taiwan

## Abstract

Glutathione S-transferases (GSTs) are multifunctional proteins encoded by large gene family in plants, which play important role in cellular detoxification of several endobiotic and xenobiotic compounds. Previously, we suggested the diverse roles of rice GST gene family members in plant development and various stress responses based on their differential expression. In this study, we report the functional characterization of a rice tau class GST gene, *OsGSTU4*. OsGSTU4 fusion protein was found to be localized in nucleus and cytoplasm. The over-expression of *OsGSTU4* in *E. coli* resulted in better growth and higher GST activity under various stress conditions. Further, we raised over-expression transgenic Arabidopsis plants to reveal its *in planta* function. These transgenic lines showed reduced sensitivity towards plant hormones, auxin and abscisic acid. Various analyses revealed improved tolerance in transgenic Arabidopsis plants towards salinity and oxidative stresses, which may be attributed to the lower accumulation of reactive oxygen species and enhanced GST activity. In addition, microarray analysis revealed up-regulation of several genes involved in stress responses and cellular detoxification processes in the transgenic plants as compared to wild-type. These results suggest that *OsGSTU4* can be used as a good candidate for the generation of stress-tolerant crop plants.

## Introduction

Abiotic stresses (salinity, drought, oxidative and osmotic stresses) are the major environmental factors affecting crop production worldwide by reducing growth and productivity of plants. Exposure of plants to various abiotic stresses leads to oxidative damage [1]. Plants employ various defense mechanisms to overcome these damages [2]. The study of plant tolerance towards abiotic stresses, in order to identify and modify the genes involved in abiotic stresses, seems to be a promising approach to generate stress-tolerant plants.

Glutathione S-transferases (GSTs) are ubiquitous enzymes encoded by a large family of genes, which play an important role in cellular detoxification to a wide variety of endobiotic and xenobiotic substrates by conjugating the tripeptide glutathione [3]–[7]. Several GSTs have been identified and analyzed in different organisms including plants, animals, fungi and bacteria [8], [9]. On the basis of predicted amino acid sequences, the soluble GSTs have been divided into the phi, tau, lambda, dehydroascorbate reductase (DHAR), theta, zeta and elongation factor 1 gamma (EF1G) classes. Among these, phi, tau, lambda and DHAR classes are specific to plants [10]. Previously, we identified a total of 79 GST genes in the rice genome and classified them in various families [11]. On the basis of protein sequence alignment and evolutionary relationship, fifty two (52) GST genes were included in tau, 17 in phi, 4 in zeta, two in DHAR, two in EF1G and one each in theta and tetrachlorohydroquinone dehalogenase (TCHQD) classes.

GSTs have been found to be differentially regulated by various abiotic stresses, including dehydration [12], herbicide [10], wounding [13], cold [14], hypoxic [15], H_2_O_2_ [16], UV-light [17], phosphate starvation [18], metals [19] and salt stress [20], [21]. Studies have shown that expression of GSTs is activated by biotic stresses, such as pathogen attack and fungal elicitors too [3]. The expression of GSTs is induced by plant hormones, such as ethylene [22], auxin [23], methyl jasmonate and salicylic acid [24] and ABA [25]. Several studies have shown that GST activity in plants protect the cells from various types of abiotic and biotic stresses [17], [19], [26], [27], [28]. From a recent study, it was found that plant tau class GSTs have been involved in regulation of cell elongation and flowering in response to light [29]. Over-expression of GST genes into low-temperature sensitive rice plants enhanced their tolerance to low temperature [30] and in tobacco plants increased the resistance to oxidative stresses [31]. In a recent study, Chen et al. [29] reported that AtGSTU20 physically interacts with far-red insensitive 219 (FIN219) protein in response to light and plays a crucial role in cell elongation and plant development. Co-expression of GST and CAT1 genes in rice provided increased tolerance to oxidative stress [32]. Over-expression of cotton GST enhanced tolerance to oxidative stresses in tobacco plants [31].

In our previous study, we observed preferential expression of GST genes in various developmental stages, and differential expression in response to various stresses [11]. Although several GSTs have been identified in rice, only a few of them have been functionally characterized as of now. In this study, we characterized the role of a tau class GST gene from rice, *OsGSTU4* (Genbank accession number FN428745). We raised and analyzed Arabidopsis transgenic plants over-expressing *OsGSTU4*. Our results showed increased tolerance in the transgenic plants to salinity and oxidative stresses at various developmental stages.

## Materials and Methods

### Plant material and growth conditions

For gene expression analysis in rice, tissue samples from various developmental stages, hormone treatments, and abiotic stress conditions were used as described previously [11]. *Arabidopsis thaliana* ecotype Columbia was used as wild-type (WT) for all experiments and genetic background for the generation of transgenic plants. Seeds were obtained from Arabidopsis Biological Resource Centre, USA. Plants were grown in plates containing MS medium and in pots containing vermiculite supplemented with liquid Arabidopsis nutrient medium in a culture room under 80–100 μmol m^−2^ S^−1^ at 22±1°C with 14/10 h of day/night photoperiod, as described previously [33].

### Bacterial over-expression and protein purification

For over-expression in *Escherichia coli*, the complete open reading frame (ORF) of *OsGSTU4* was amplified by PCR using gene-specific primers (TAAGGATCCATCCATGGCCGGTGGAG and GCAAAGCTTTCAGTTGGTGGCAG) and cloned into the expression vector, pET28a, in *Bam*HI and *Hin*dIII restriction sites. The resulting recombinant plasmid, *pET28a:OsGSTU4* was transformed into *E. coli* strain BL21 (DE3) pLysS. For the expression of OsGSTU4 protein, *E. coli* cells were induced by addition of 1 mM isopropyl β-D-1-thiogalactopyranoside (IPTG) at 37°C. Induction of OsGSTU4 protein was analyzed in crude protein extracts by polyacrylamide gel electrophoresis (PAGE). Induced protein was purified using nickel-charged affinity column under native conditions as per manufacturer's protocol (Qiagen GmbH, Hilden).

### Growth response assay and GST activity measurement in *E. coli*

*E. coli* cells transformed with empty vector (pET28a) and *pET28a:OsGSTU4* were cultured overnight at 37°C and inoculated in fresh Luria-Bertani (LB) medium. After obtaining OD_600_ of 0.4–0.6, the expression of OsGSTU4 protein was induced by adding 1 mM IPTG and bacterial cultures were allowed to grow with or without 1 mM methyl viologen (MV), 1 mM hydrogen peroxide (H_2_O_2_), 300 mM mannitol and 300 mM sodium chloride (NaCl). After 3–4 h of incubation, growth of cultures was measured using spectrophotometer (Shimadzu 300, Shimadzu Corporation, Tokyo, Japan). For measurement of GST activity, total protein was extracted from bacterial cultures by resuspending the pellet in lysis buffer supplemented with 1 mM phenylmethanesulfonyl fluoride (PMSF) and 2 mg/ml lysozyme followed by sonication. The supernatant was used to measure GST activity in pET28a and *pET28a:OsGSTU4* transformed cells under non-stressed and stressed conditions using 1-chloro-2, 4-dinitrobenzene (CDNB) as a substrate, as described earlier [34]. The activity was determined spectrophotometrically by measuring change in absorbance at 340 nm.

### Sub-cellular localization

For transient expression in onion epidermal cells, a *psGFPcs:OsGSTU4* construct was made by fusing the complete ORF of *OsGSTU4* in psGFPcs vector [35] in *Apa*I and *Xma*I restriction sites. psGFPcs is a green fluorescent protein (GFP) tag containing vector used in this study to visualize the localization of OsGSTU4. The empty vector (*psGFPcs*) and fusion construct (*psGFPcs:OsGSTU4*) were transformed on onion peels by particle bombardment method as described previously [36]. Both the plasmid constructs were transformed on Arabidopsis leaves also by biolistic method using Helios Gene Gun (Bio-Rad) as per earlier described protocol [37]. The onion peels and Arabidopsis leaves were visualized using confocal fluorescence microscopy (AOBS TCS-SP2, Leica Microsystems, Mannheim) to detect GFP and/or DAPI signals after 24 h of incubation.

### Over-expression construct and transformation in Arabidopsis

The complete ORF of *OsGSTU4* was PCR amplified using gene-specific primers (TATTCTAGAATGGCCGGTGGAGGA and TATGGATCCTCAGTTGGTGGCAG) and cloned in pBI121 vector in *Xba*I and *Bam*HI restriction sites. Further, *pBI121:OsGSTU4* construct was confirmed by restriction digestion and sequencing and transformed into *Agrobacterium* strain GV3101. Agrobacterium-mediated transformation of Arabidopsis plants was performed via floral dip method [38]. Primary transformants were screened on MS medium containing kanamycin antibiotic selection and grown till homozygous stage (T_4_ and T_5_ generation) as described previously [33] for further analysis.

### Root growth inhibition and seed germination assays

To assay the response of WT and Arabidopsis transgenic seedling roots towards plant hormones, auxin (IAA) and abscisic acid (ABA), and abiotic stresses (salinity and oxidative), seedlings were grown vertically on Petri plates containing MS medium. After 5 days of growth, seedlings were transferred onto fresh MS medium supplemented with various concentrations of plant hormones (0.1, 0.5 μM IAA and 5, 10 μM ABA) and abiotic stresses (150 mM NaCl, 1 μM MV and 4 mM H_2_O_2_) and root growth of WT and transgenics were measured as described earlier [33]. For germination assay, seeds were plated on MS medium supplemented with 150 mM NaCl, 1 μM MV and 5 mM H_2_O_2_. Percentage of germination was recorded after 5 days as described previously [36].

### Leaf disc assays and estimation of chlorophyll content

To assess the effect of salinity and oxidative stresses on the leaves of transgenic Arabidopsis and WT plants, leaf disc assays were performed under control (water) conditions or in the presence of 100 mM and 200 mM NaCl for salinity stress and 5 μM and 10 μM MV for oxidative stress as described previously [36]. Chlorophyll content was measured from the non-stressed and stressed leaves by keeping them overnight in dark in 80% acetone. Absorbance was recorded at 645 and 663 nm using a UV-Vis spectrophotometer (Shimadzu 3000, Shimadzu Corporation, Tokyo). The total chlorophyll content was measured and normalized per gram of fresh weight sample as described [36].

### Phenotypic evaluation of transgenics under stress conditions

To evaluate tolerance towards salinity and oxidative stresses, WT and transgenic Arabidopsis seedlings were grown in Petri plates. Seedlings with uniform growth for WT and transgenic lines were transplanted into plastic pots after 15 days. Stress tolerance assay was performed on one-month-old Arabidopsis seedlings by irrigating them with 200 mM NaCl for salinity and 25 μM MV for oxidative stress. Phenotypic and growth parameters, such as plant height (growth), biomass and survival rate were scored after imposing one week of stress treatments.

### Reactive oxygen species (ROS) detection and measurement of GST activity

To detect ROS levels in WT and transgenic Arabidopsis plants, we estimated H_2_O_2_ and superoxide (O^2**•–**^) levels. The detached leaves from WT and transgenic Arabidopsis plants grown under non-stressed (water), salinity (200 mM NaCl) and oxidative (25 μM MV) stress conditions, were placed in 1 mg/ml solution (pH 3.8) of 3, 3′-diaminobenzidine (DAB) for detection of H_2_O_2_ and nitro blue tetrazolium (NBT) solution for O^2**•–**^ and incubated at room temperature for 4 h in dark. After staining, accumulation of H_2_O_2_ and O^2**•–**^ were detected as brown color precipitate and blue spots, respectively. The stained leaves were kept in absolute ethanol for 4 h to remove chlorophyll. Subsequently, the bleached leaves were transferred in 70% ethanol and photography was performed.

GST activity in the protein extracts of WT and transgenic plants under non-stressed, salinity and oxidative stress conditions was measured as described above. The estimation was carried out from at least three independent biological replicate samples.

### Real time PCR analysis

Total RNA was isolated from transgenic Arabidopsis seedlings by using TRI reagent according to manufacturer's instructions (Sigma Life Science, USA). First strand cDNA was synthesized using 1 μg total RNA. Real time PCR analysis was performed in three biological replicates as described previously [36] using gene-specific primers. For gene expression analysis in rice during various developmental stages, hormone and stress conditions, tissue and RNA samples were prepared as described previously [11]. The sequences of primers used for real-time PCR analysis are listed in Table S1. The *UBQ5* and *PP2A* genes were used as internal control for gene expression data analysis in rice and Arabidopsis, respectively.

### Microarray analysis

For microarray analysis, transgenic and WT plants were grown for two weeks under normal growth condition and three independent biological replicates were harvested. RNA isolation was done using TRI reagent (Sigma), and quality and quantity of RNA were checked by Agilent Bioanalyzer (Agilent Technologies, North Carolina, USA). Microarray experiment was performed using Affymetrix GeneChip 3′-IVT express kit according to manufacturer's instructions (Affymetrix, Santa Clara, USA) as described previously [36]. Gene ontology (GO) and metabolic pathway enrichment analyses were performed using BiNGO and AraCyc database, respectively, as described earlier [39]. The heatmaps showing the expression profiles of genes were generated using signal intensity values via MultiExperiment Viewer (v4.8). Microarray data have been submitted to Gene Expression Omnibus database under the series accession number GSE53152.

### Statistical analysis

Data of 10–12 seedlings for root growth measurement, 40–60 seeds for seed germination assay, four to five leaves for leaf disc assays and 10–12 plants for phenotypic evaluation from WT and transgenic plants were collected. Each experiment was repeated at least three times. Mean value and standard error (SE) were calculated. Student's t-test was performed to reveal significant differences between WT and transgenic lines. A *P*-value of ≤0.05 was considered significant.

## Results

### Differential accumulation of *OsGSTU4* transcripts

**Figure 1:**
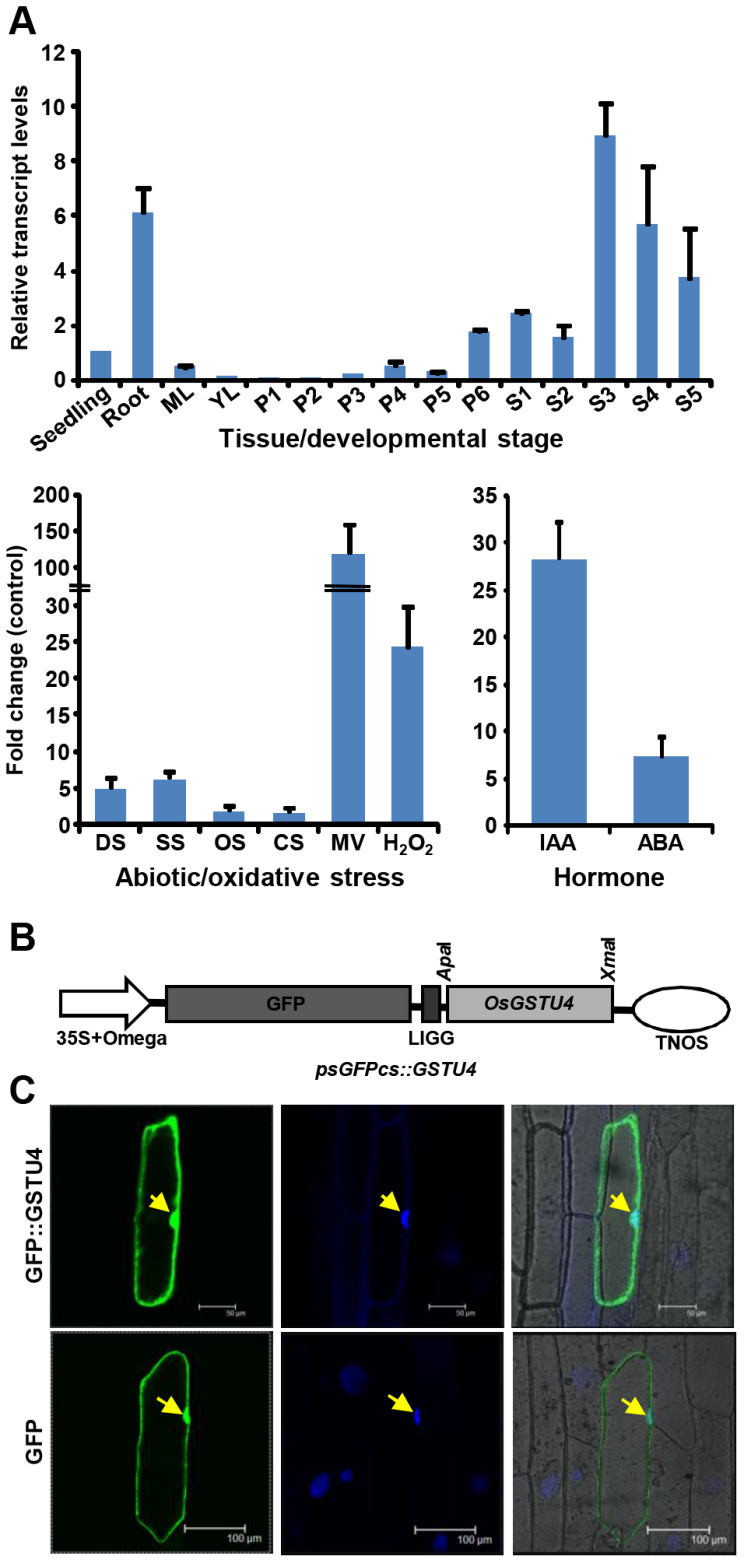
Gene expression analysis and sub-cellular localization of OsGSTU4. (A) Real-time PCR analysis showing transcript levels of *OsGSTU4* in various tissues/developmental stages (ML, mature leaf; YL, Y leaf; P1-P6 stages of panicle development; S1–S5, stages of seed development) and under hormone (IAA, auxin; ABA, abscisic acid), abiotic stress (DS, dehydration; SS, salinity; OS, osmotic; CS, cold_ and oxidative stress (MV, methyl viologen; H_2_O_2_, hydrogen peroxide) treatments. The relative mRNA levels as compared to seedling (for tissues/developmental stages) and control mock-treated samples (hormone and stress treatments) have been presented. (B) The *psGFPcs::GSTU4* construct used for transient expression in onion epidermal cells under the control of CaMV 35S promoter. (C) Images were observed in GFP filter (*left*), after DAPI staining (*middle*) and merged (*right*) by confocal microscopy after 24 h of incubation. In *psGFPcs:OsGSTU4*, GFP signal was detected in the nucleus and cytoplasm, whereas for psGFPcs (empty vector), GFP signal was detected in the whole cell. Arrows represent location of the nuclei.

In our previous study, we observed differential expression of several GST gene family members in the presence of various plant hormones, and abiotic and biotic stresses; and majority of them belonged to tau class, based on microarray data analysis [11]. The transcript level of one of the tau class GST gene, *OsGSTU4*, was induced by dehydration, salt and cold stresses. This gene was found to be preferentially expressed in the root and seed development stages and also found to be highly up-regulated by plant hormones, abiotic and biotic stress conditions. Here, we sought to validate the role of *OsGSTU4* in various abiotic stress responses. We examined the expression of *OsGSTU4* in various tissues/developmental stages (root, mature leaf, Y leaf, and stages of panicle and seed development), and hormone (auxin, IAA and abscisic acid, ABA) and abiotic stress (dehydration, salinity, osmotic, cold and oxidative) treated samples via real-time PCR analysis (Figure 1A). We observed higher expression of *OsGSTU4* in root and various stages of seed development. We observed up-regulation of *OsGSTU4* in rice seedlings subjected to IAA and ABA treatments. Further, the transcript levels of *OsGSTU4* were elevated significantly under abiotic (salt, osmotic, desiccation and cold) and oxidative (MV and H_2_O_2_) stress conditions as well. The expression analysis of *OsGSTU4* in this study corroborated our previous observation [11].

### Sub-cellular localization of OsGSTU4

To investigate the subcellular localization of OsGSTU4, we cloned *OsGSTU4* coding sequence in psGFPcs vector and expressed the fusion protein transiently in onion epidermis cells and Arabidopsis leaf epidermal cells under the control of 35S promoter (Figure 1B). The GFP fusion protein of OsGSTU4 was found to be localized in nucleus and cytoplasm (Figure 1C, Figure S1). However, GFP alone was found to be distributed in the whole cell. The N-terminal fusion may not affect the localization of OsGSTU4. Previous studies have revealed the localization of other proteins in different cell compartments via C-terminal fusion using the same vector [35], [36].

### Over-expression of *OsGSTU4* improves tolerance to various stresses in *E. coli*

**Figure 2:**
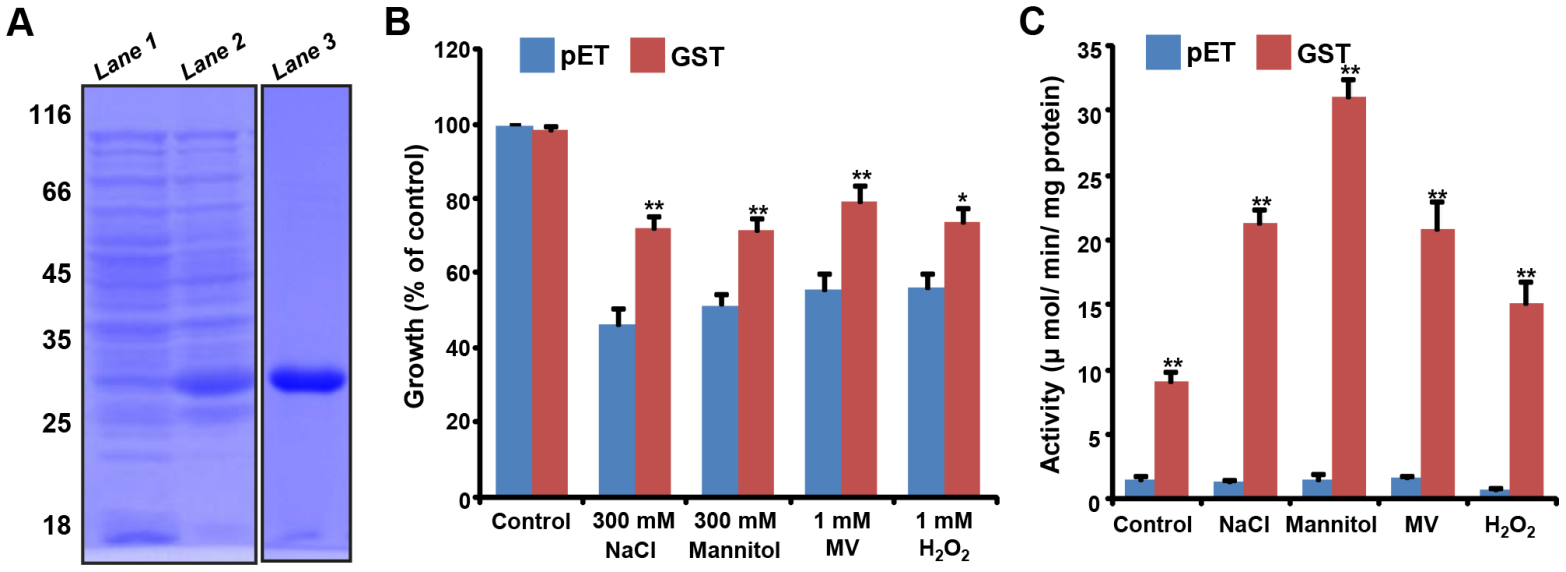
Expression of *OsGSTU4* in *E. coli* and evaluation of stress responses. (A) SDS-PAGE (12%) analysis showing induction and purification of 6xHis:OsGSTU4 protein (*Lane 1*, *OsGSTU4* transformed uninduced cells; *lane 2*, *OsGSTU4* transformed cells induced with 1 mM IPTG; *lane 3*, 6x:His:OsGSTU4 purified (∼25.5 kDa) protein). (B, C) Growth and GST activity of *E. coli* cells under various stresses (mentioned below each bar). (B) Growth of *E. coli* cells into empty vector (pET) and *pET28a:OsGSTU4* (GST) transformed cells under different stress treatment. (C) GST activity in crude soluble protein, extracted from pET28a (pET) and *pET28a:OsGSTU4* (GST) transformed *E. coli* cells under different stress conditions. Percentage of growth and activity under stress conditions has been given relative to the percentage of growth under control condition. Data were obtained from three independent replicates and are means ± SE. Data points marked with asterisk (*P≤0.05 and **P≤0.01) indicate statistically significant differences.

The complete ORF of *OsGSTU4* was cloned in pET28a vector and expressed in *E. coli* to check the response to various stresses. After induction with 1 mM IPTG, protein extracts were analyzed by SDS-PAGE. The analysis showed induction of ∼25 kDa protein in *pET28a:OsGSTU4* transformed cells, which was absent in uninduced cells (Figure 2A). Solubility analysis revealed that most of the induced protein was present in the supernatant (soluble fraction). The 6xHis:OsGSTU4 protein was purified from the soluble fraction to homogeneity using nickel-charged affinity column (Figure 2A).

To understand the role of *OsGSTU4* in stress protection, we investigated the growth pattern of *E. coli* transformed with *pET28a:OsGSTU4* or empty vector under various stress conditions. We did not observe any significant difference in the growth of *pET28a:OsGSTU4* and pET28a transformed cells under non-stressed condition. However, better growth of *pET28a:OsGSTU4* transformed cells was observed under various stress conditions, including salinity (26%), osmotic (20%) and oxidative (MV, 24% and H_2_O_2_, 18%) stresses (Figure 2B). Further, we measured the GST activity in the crude protein samples extracted from *pET28a:OsGSTU4* and pET28a transformed cells. The results showed higher GST activity in *pET28a:OsGSTU4* transformed cells as compared to the pET28a transformed cells under different stress conditions analyzed (Figure 2C). These results suggested that over-expression of OsGSTU4 improves stress tolerance in *E. coli*.

### Over-expression of *OsGSTU4* in Arabidopsis plants

**Figure 3:**
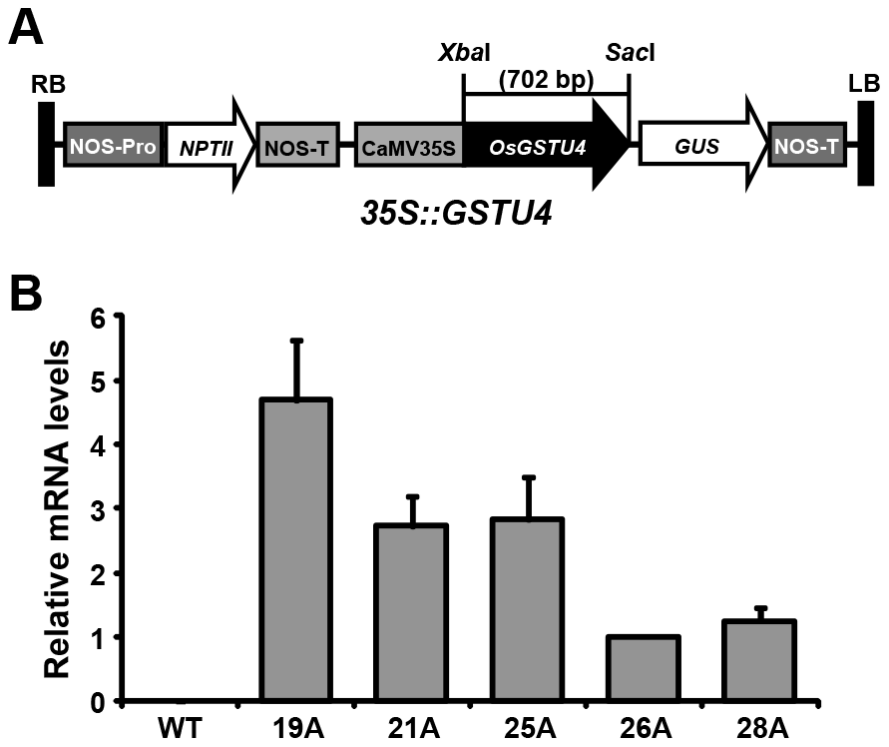
Over-expression of *OsGSTU4* in transgenic Arabidopsis plants. (A) Schematic representation of *35S:OsGSTU4* construct used for over-expression in Arabidopsis. The complete ORF of *OsGSTU4* was cloned in pBI121 vector carrying 35S promoter (B) Real-time PCR analysis of WT and transgenic Arabidopsis plants, showing the expression of *OsGSTU4* in five homozygous transgenic lines (19A, 21A, 25A, 26A and 28A) with no detectable expression in WT. The expression of *OsGSTU4* in homozygous transgenic lines are shown relative to 26A line exhibiting the lowest expression of the transgene. The expression of *PP2A* gene was used as internal control. Data are mean ± SE from three biological replicates.

To study the role *OsGSTU4 in planta*, we expressed *OsGSTU4* in Arabidopsis plants under the control of CaMV 35S promoter (Figure 3A). Fifteen kanamycin-resistant plants were obtained via floral-dip transformation and grown to the homozygous stage as described previously [36]. Five transgenic lines, 19A, 21A, 25A, 26A and 28A were selected for further analysis. PCR analysis confirmed the presence of transgene in the transgenic plants. All the obtained transgenic lines showed no significant phenotypic/growth differences as compared to WT under normal growth conditions (Figure S2A, B). Similar type of phenotype in transgenic Arabidopsis and WT, indicates that overexpression of *OsGSTU4* does not affect growth and development of Arabidopsis plants. Similar result was observed in transgenic tobacco [20] and transgenic Arabidopsis plants also [36]. Over-expression of the *OsGSTU4* in transgenic plants was confirmed by real-time PCR analysis. All the transgenic lines showed the expression of transgene with varied transcript levels as compared to no detectable expression in WT (Figure 3B). Three transgenic lines (21A, 26A and 28A) were selected for further analysis on the basis of different levels of transgene expression and enough seed availability.

### Reduced sensitivity of *OsGSTU4* over-expression transgenic Arabidopsis plants to auxin and ABA

**Figure 4:**
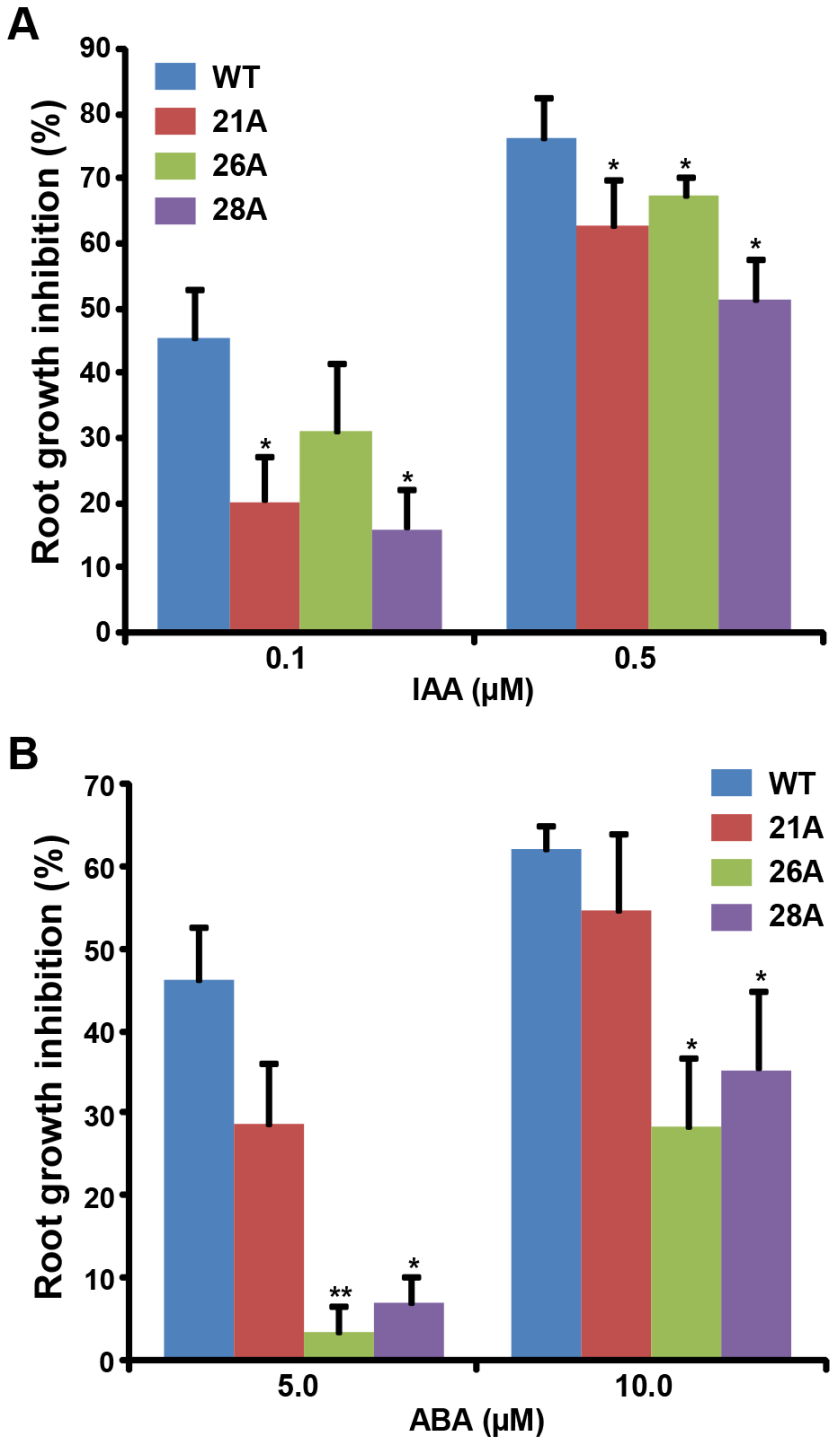
Effect of auxin and stress hormone ABA on transgenic plants. Reduced root growth inhibition (A, B) of *35S:OsGSTU4* transgenic lines in the presence of IAA (A) and ABA (B). Root length of transgenic Arabidopsis and WT seedlings were measured after 5 days of growth on MS medium or IAA and ABA supplemented MS medium. Root growth inhibition of IAA and ABA treated seedlings was expressed as percentage of control incubated on IAA- and ABA-free medium. The experiment were repeated at least three times and values presented are mean ± SE. Data points marked with asterisk (*P≤0.05 and **P≤0.01) indicate statistically significant difference between WT and transgenic lines.

To examine the response of transgenic plants with respect to plant hormones, IAA and ABA, root growth inhibition assays were performed. The root growth of transgenic and WT seedlings on various concentrations of IAA (Figure 4A) and ABA (Figure 4B) was analyzed. We found better root growth for transgenic plants after 5 days of growth as compared to WT. The inhibition in root growth of different transgenic lines was 14–29% and 9–25% lower in the presence of 0.1 μM and 0.5 μM IAA, respectively. Likewise, transgenic plants showed lesser root growth inhibition on 5 μM (18–42% lesser) and 10 μM (5–30% lesser) on ABA as compared to WT (Figure 4B). These observations suggested lower sensitivity of transgenic Arabidopsis plants towards auxin and stress hormone ABA.

### Enhanced salinity and oxidative stress tolerance of *OsGSTU4* over-expression transgenic Arabidopsis plants

**Figure 5:**
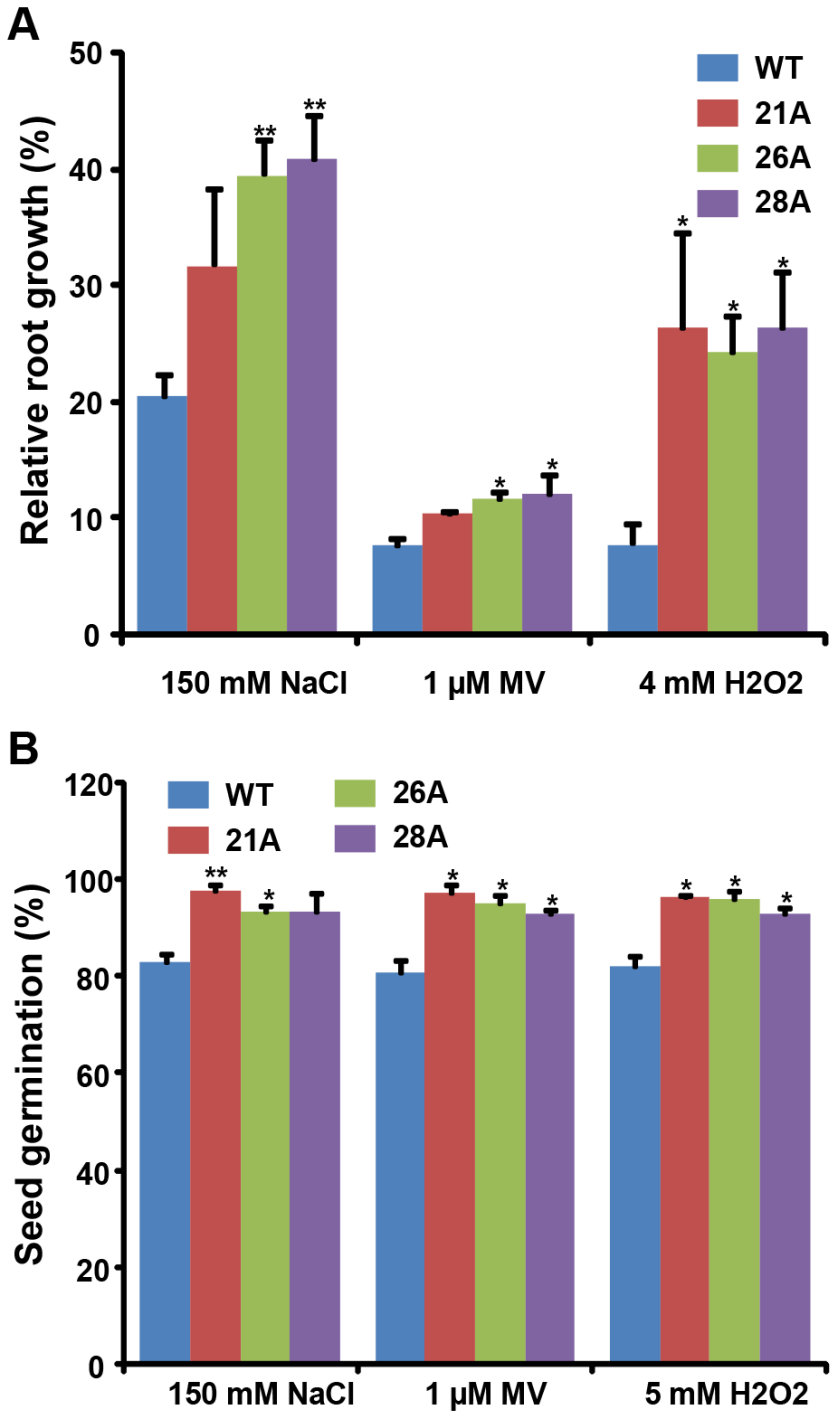
Root growth and seed germination assays showing response of WT and OsGSTU4 over-expression transgenic plants to salinity and oxidative stress. Comparison of root growth (A) and seed germination (B) of WT and transgenic Arabidopsis lines under salinity and oxidative stresses. (A) Increased percentage of relative root growth in transgenic Arabidopsis seedlings in response to salinity (150 mM NaCl) and oxidative (1 μM MV and 4 mM H_2_O_2_) stress. Root growth of seedlings under stress conditions was expressed as percentage of controls incubated on MS medium. (B) Enhanced seed germination in Arabidopsis transgenic lines expressing *OsGSTU4* in the presence of 150 mM NaCl, 1 μM MV and 5 mM H_2_O_2_. The number of germinated seeds was expressed as the percentage of total number (40–60) of seeds plated. All the experiments were repeated three times and values are mean ± SE. Significance analysis was done by Student's t-test (*P≤0.05, **P≤0.01).

Further, we analyzed the response of transgenic and WT plants to various abiotic stresses. In root growth assays, we observed increased tolerance in roots of transgenic lines under salinity and oxidative stresses. The root growth of transgenic lines was better under salinity and oxidative stress as compared to WT. The root length of transgenic seedlings was significantly higher in the presence of 150 mM NaCl (11–20%), 1 μM MV (3–5%) and 4 mM H_2_O_2_ (15–17%) (Figure 5A). In seed germination assays also, we found reduced sensitivity of transgenic lines at 150 mM NaCl, 1 μM MV and 5 mM H_2_O_2_ (Figure 5B) as compared to WT plants. The seed germination was higher on 150 mM NaCl (10–15%), 1 μM MV (11–15%) and 5 mM H_2_O_2_ (11–14%) in different transgenic lines. However, we did not find any difference due to osmotic stress in seed germination and root growth under various concentrations of mannitol (data not shown).

**Figure 6:**
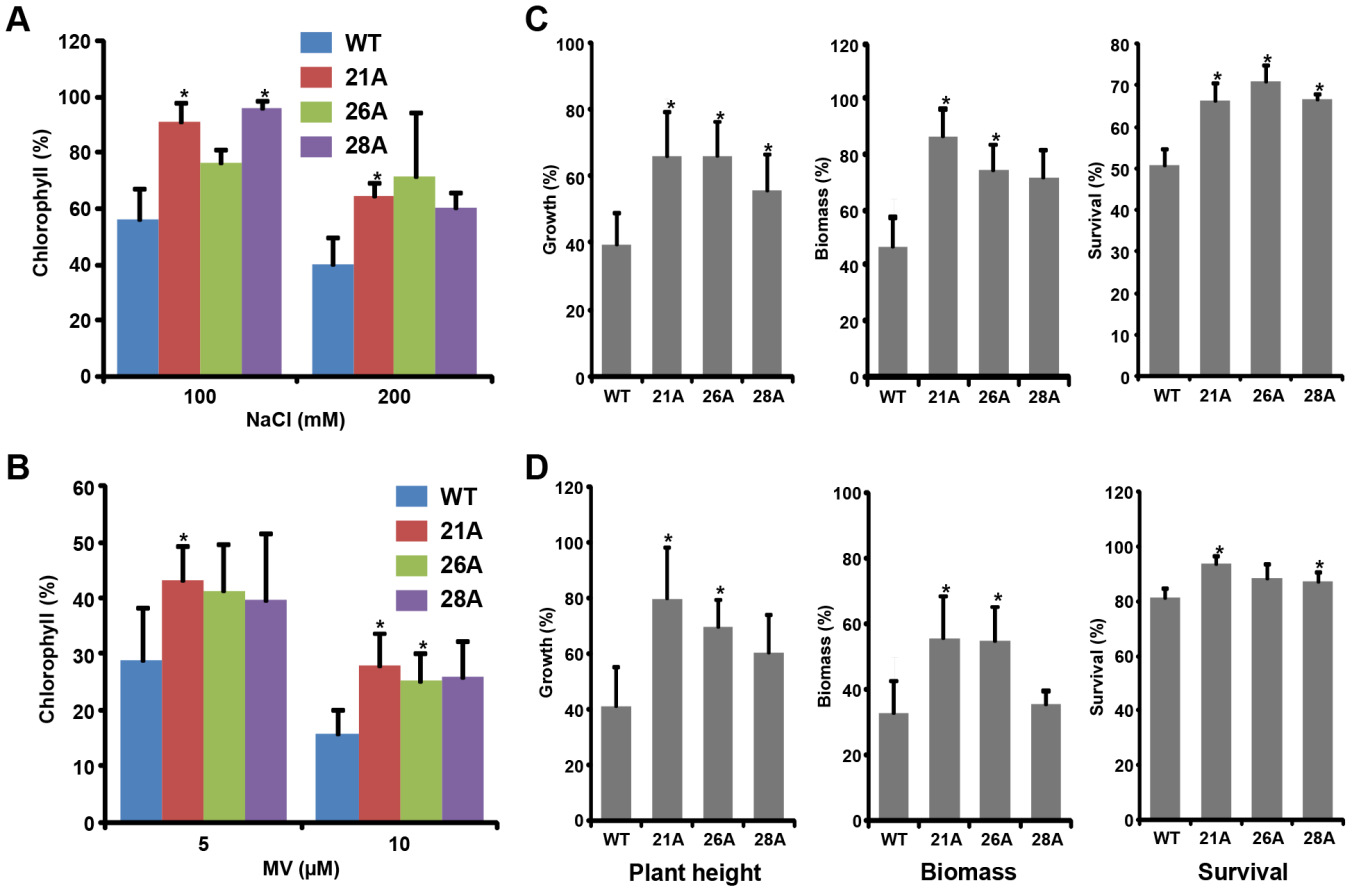
Effect of salinity and oxidative stresses on *OsGSTU4* over-expression Arabidopsis transgenic lines. Comparison of chlorophyll content (A, B), plant height, biomass and survival rate (C, D) under salinity (A, C) and oxidative (B, D) stress conditions. (A, B) The chlorophyll content in WT and transgenic lines under stress condition are shown relative to the chlorophyll content under control condition. (C, D) Various phenotypic parameters of transgenic Arabidopsis and WT plants in the presence of 200 mM NaCl (C) and 25 μM MV (D) have been given. Percentage of growth (plant height), biomass and survival under salinity and oxidative stress condition was expressed as the percentage of plant growth under control condition. Values are mean ± SE from three independent experiments. Data points marked with asterisk (*P≤0.05) indicate statistically significant difference between WT and transgenic lines.

To further confirm the salinity and oxidative stress tolerance of *OsGSTU4* expression transgenic plants, leaf disc assays were performed in the presence of various concentrations of NaCl and MV. The results showed less chlorosis in the leaves of transgenic plants as compared to WT under stress conditions (Figure S3). This stress-tolerant phenotype was further confirmed quantitatively by detection of higher levels of chlorophyll content in transgenic plants under salinity (Figure 6A) and oxidative stress (Figure 6B) conditions. Under non-stressed condition, chlorophyll content and phenotype was similar in both, WT and transgenic lines. However, with increasing concentration of salinity and oxidative stresses, both transgenic and WT plants showed higher chlorosis, having lower chlorophyll content as compared to the control conditions, but the effect was more severe in WT plants. During salinity stress, chlorophyll content was (19–32%) higher at 100 mM NaCl and (21–32%) higher at 200 mM NaCl in transgenic plants as compared to WT. In the presence of oxidative stress, the percentage of chlorophyll was higher at 5 μM (7–9%) and 10 μM (6–8%) MV in transgenic plants as compared to WT.

Further, to measure the tolerance of mature transgenic plants towards various abiotic stresses, one month old plants of three transgenic lines, 21A, 26A and 28A, along with WT, were irrigated with water (control), 200 mM NaCl (salinity stress) and 25 μM MV (oxidative stress). There were no phenotypic differences between WT and transgenic lines under control conditions. However, transgenic plants showed tolerant phenotype (remained green and less growth retardation) and were healthier than WT under stress conditions. After treatment, WT plants showed more wilting as compared to transgenic lines. After one week of treatments, phenotypic features (height of the plants (growth), biomass (plant weight) and survival rate) were recorded. These parameters further confirmed the tolerant phenotype of transgenic lines at quantitative level. During salinity stress (200 mM NaCl), we observed tolerant phenotype (Figure S4A) in transgenic plants with (16–27%) higher plant growth, (28–46%) higher biomass and better survival rate in different transgenic lines as compared to WT (Figure 6C). Under oxidative stress (25 μM MV) condition also, both the WT and transgenic plants showed growth retardation, however the extent of retardation was lesser in transgenics as compared to WT. We observed stress tolerant phenotype (Figure S4B), better growth (19–39% higher), increased biomass (21–28% higher) and increased survival rate in different transgenic lines as compared to WT (Figure 6D). Stress tolerant phenotype (both phenotypic and quantitative level) of Arabidopsis transgenic plants, suggested that the over-expression of *OsGSTU4* in Arabidopsis increased the tolerance to salinity and oxidative stresses.

### GST enzyme activity and ROS level in Arabidopsis transgenic lines

**Figure 7:**
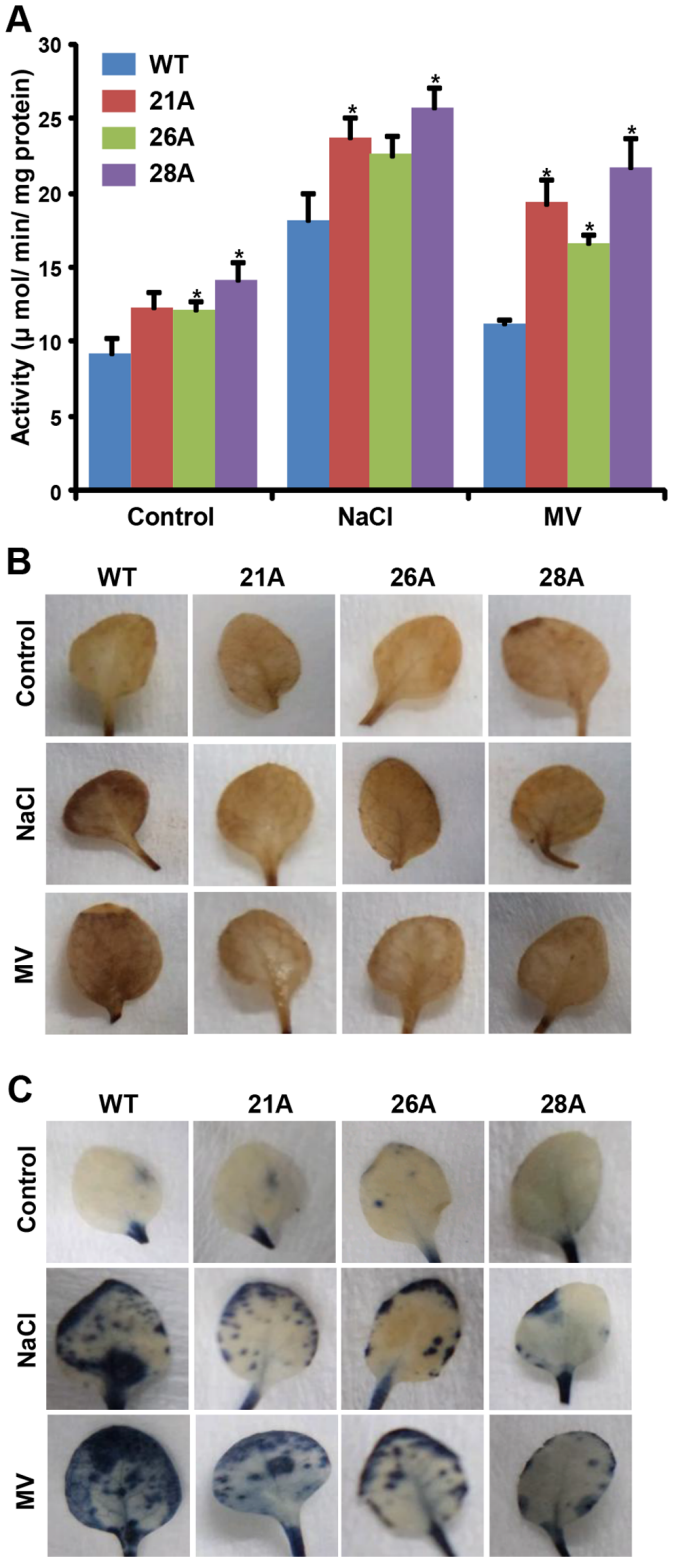
GST activity and ROS levels of WT and transgenic plants during salinity and oxidative stresses. GST activity (A), H_2_O_2_ (B) and O^2**•–**^ (C) levels in transgenic Arabidopsis and WT plants under salinity and oxidative stress condition. (A) Graph showing increased GST activity in transgenic Arabidopsis lines expressing *OsGSTU4* in the presence of 200 mM NaCl and 25 μM MV. (B, C) Decreased ROS [H_2_O_2_ (B) and O^2**•–**^ (C)] level in the leaves of transgenic Arabidopsis as compared to WT in response to salinity and oxidative stress. The experiments were repeated at least three times and values are mean ± SE. Data points marked with asterisk (*P≤0.05) indicate statistically significant differences between WT and transgenic lines.

To investigate the potential mechanism of improved stress tolerance, we measured the GST activity and ROS levels in transgenic *Arabidopsis* and WT plants. The results showed that GST activity was slightly higher in the transgenics as compared to WT plants and was comparable among the transgenic lines under control conditions (Figure 7A). Further, it was found that GST activity was significantly enhanced in the transgenics under salinity and oxidative stress conditions as compared to WT plants (Figure 7A).

ROS (H_2_O_2_ and O^2**•–**^) levels were also measured in leaves of transgenic and WT plants, respectively, under non-stressed and stressed conditions. The leaves of WT plants showed higher level of H_2_O_2_ (Figure 7B) and O^2**•–**^ (Figure 7C) under salinity and oxidative stress conditions, respectively. However, lower accumulation of H_2_O_2_ and O^2**•–**^ was observed in transgenic plants during salinity and oxidative stress conditions (Figure 7B, C), which further confirmed the stress tolerance of OsGSTU4 over-expressing plants. Together, these results suggest that the improved tolerance of transgenic lines to salinity and oxidative stresses might be, at least in part, due to lower accumulation of ROS.

### Global gene-expression analysis in transgenic Arabidopsis

**Figure 8:**
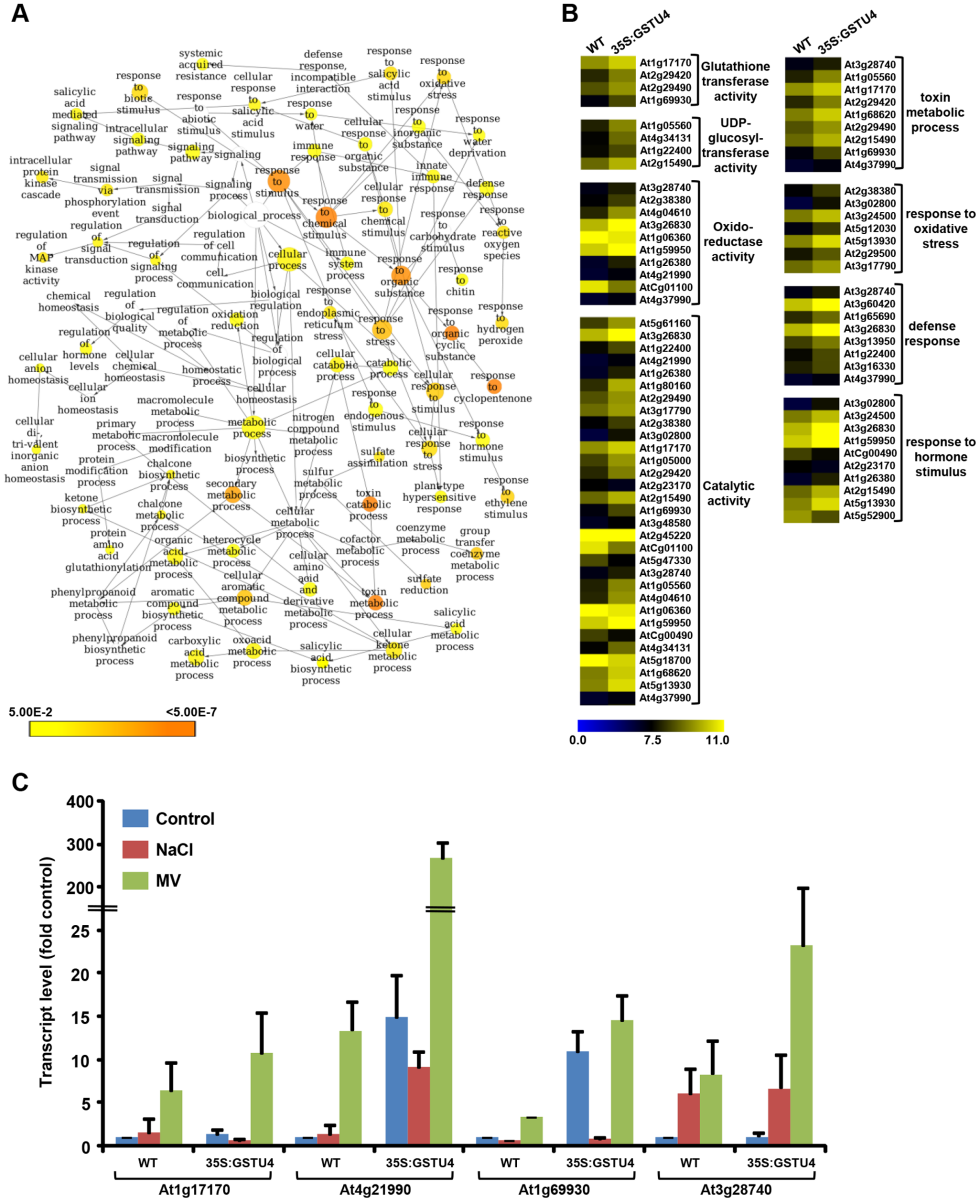
Differential gene expression in over-expression transgenic lines. (A) Significantly enriched GO biological process terms in the up-regulated genes. Node size is proportional to the number of transcripts in each category and colors shaded according to the significance level (white - no significant difference; color scale, yellow – *P*-value  = 0.05, orange – *P*-value <0.0000005). (B) Heatmaps show the differential expression of genes involved in various biological processes and molecular functions found enriched in genes up-regulated in transgenic line. The color scale at the bottom represents the log transformed signal intensity. (C) Real-time PCR analysis showing differential expression of selected genes in transgenic lines under control, and stress conditions. The graph displays the fold change in mRNA levels in WT and transgenic line under control and stress (NaCl and MV) conditions as compared to the WT-control.

To study the effect of *OsGSTU4* over-expression on global gene expression in Arabidopsis, microarray analysis of WT and transgenic line (26A) grown under normal growth condition was performed. The data analysis identified a total of 63 genes (45 up-regulated and 18 down-regulated) significantly (fold change ≥2.0 with *P*-value ≤0.05) differentially expressed in the transgenic line as compared to WT (Table S2). Broadly, these genes encode for proteins involved in stress responses, transcription factors, photosynthesis and cellular detoxification processes. Many of these genes were well-known stress-responsive genes. Further, we performed GO enrichment analysis to identify the biological processes significantly altered in the transgenic plants. We found that GO terms, toxin metabolic processes, response to oxidative stress, defense response, sulfate reduction and response to hormone stimulus were significantly enriched in the up-regulated genes in transgenic plants (Figure 8A, B). Among the molecular function GO terms, glutathione-mediated detoxification, UDP-glucosyltransferase activity, catalytic activity and oxidoreductase activity were significant in the up-regulated genes (Figure 8B). Many of these biological processes and molecular function terms have been implicated in abiotic stress responses. Further, we also analyzed the metabolic pathways significantly enriched in the differentially expressed in transgenic plants. The genes involved in metabolic processes, such as glutathione detoxification, sulfate reduction, phenylpropanoid derivative biosynthesis and flavonoid biosynthesis pathways were found significant, which was consistent with GO enrichment analysis. In addition, we analyzed the transcript levels of putative GST and antioxidant enzyme encoding genes in WT and transgenic line under control and stress (salinity and oxidative) conditions. We observed up-regulation on these genes in the transgenic line under control conditions (Figure 8C), which corroborated the microarray results. Further, the transcript levels of these genes were up-regulated under stress conditions in both WT and transgenic lines and a higher degree of transcript up-regulation was observed in the transgenic line under stress conditions (Figure 8C).

## Discussion

Several GSTs have been identified and analyzed in plants, animals, fungi and bacteria to date [8], [9]. Plant GSTs are represented by a large multigene family and have been implicated in various stresses [11], [24], [26], [40]. Dixon and colleagues [41] reported the functionality of most of Arabidopsis GSTs in *E. coli* and suggested the functional redundancy among them to perform similar functions in different subcellular compartments. In our previous study, we identified 79 putative GSTs in rice genome, many of them showed differential expression during various hormonal treatments, biotic and abiotic i.e. salt, desiccation and cold stresses [11]. The members of *Tau* class GSTs appeared to show response to various stimuli more frequently [11]. Although several studies have reported differential expression of plant GSTs under abiotic stress conditions, only a few reports have demonstrated their role in abiotic stress tolerance [20], [32], [42].

In this study, we selected a tau class GST gene, *OsGSTU4*, on the basis of its preferential expression in response to plant hormones and differential expression in various abiotic stresses [11], and functionally characterized via over-expression in *E. coli* and Arabidopsis. The *E. coli* cells expressing *OsGSTU4* gene showed enhanced GST activity and tolerance to various abiotic stresses. The overexpression of *OsGSTU4* in Arabidopsis also imparted tolerance to salinity and oxidative stresses in transgenic plants. These results suggested the role of *OsGSTU4* in salinity and oxidative stress responses.

Several studies have implicated auxin and ABA in abiotic stress responses [36], [43]–[46]. The expression of *OsGSTU4* was found to be highly up-regulated in the presence of exogenous auxin [11]. In this study, we found reduced sensitivity of transgenic plants to exogenous auxin and ABA. Other studies have shown the effect of various abiotic stresses on auxin homeostasis as well [47]–[49]. A crosstalk between auxin and ROS signaling has been proposed and role of auxin in adaptive abiotic stress response via redox metabolism has been reported [36], [46], [50], [51]. Abiotic stresses, such as drought, salinity, cold and temperature alter the ABA levels in plants. ABA plays an important role as an essential mediator in triggering plant responses to various abiotic stresses [52]–[54]. In our study, we observed decreased root growth inhibition in transgenic plants in the presence of auxin, ABA and abiotic stresses as compared to WT. These results suggested that *OsGSTU4* might be involved in auxin- and ABA-dependent processes that provide stress tolerance to transgenic plants. OsGSTU4 may represent a component mediating cross talk between auxin and redox signaling to regulate abiotic stress responses.

The overexpression of *OsGSTU4* imparted tolerance to salinity and oxidative stresses in transgenic Arabidopsis plants. More importantly, the overexpression of OsGSTU4, did not affect the plant growth. In global transcriptome analysis, we found several stress-associated genes up-regulated in transgenic Arabidopsis plants as compared to WT under control conditions. Many of these genes are involved in cellular detoxification processes, stress responses and transcription regulation. We observed many stress related pathways, like toxin metabolic processes, response to oxidative stress, defense response, sulfate reduction, response to hormone stimulus, glutathione-mediated detoxification, UDP-glucosyltransferase activity, catalytic activity and oxidoreductase activity, enriched in the genes differentially expressed in transgenic plants. GSTs can eliminate the product of oxidative damages by the conjugation of GSH with them [55]. Earlier reports showed that CATs, GSTs and SODs play important roles in plant response to oxidative damages induced by various environmental conditions [5], [56], [57]. Salt and oxidative stress tolerance has been achieved by the co-expression of *Suaela salsa* GST and catalase (CAT) in transgenic rice [32]. In another study, overexpression of GST/GPx cDNA showed tolerance to salt and chilling stress [20] and transformation of cotton GST/GPx cDNA impart tolerance in tobacco plants to low concentration of MV [31]. Low temperature resistance was achieved by the transfer of heterologous GST gene from *Suaeda salsa* into rice [58]. In our study, we observed increased seed germination and root growth of overexpression transgenic lines in the presence of NaCl and MV. The degradation of chlorophyll in the leaves of transgenic plants was lower than the WT plants under salinity and oxidative stress conditions. Similar observations have been reported in other studies too [20], [31].

Interestingly, we observed reduced H_2_O_2_ and O^2**•–**^ accumulation in Arabidopsis transgenic plants under stress conditions. Although the transgene mRNA levels varied among the transgenic lines analyzed, the protein (GST) activity was similar under control conditions. We did not observe significant correlation between mRNA level/protein activity and response to hormones and abiotic stresses in the transgenic lines. The GST activity was found to be significantly higher in over-expressing transgenic lines under various stress conditions. The transcript level of putative GST and antioxidant enzyme encoding genes was also higher in the transgenic line. The *E. coli* cells transformed with *OsGSTU4* showed higher GST activity and thus increased tolerance to various abiotic stresses as compared to the cells transformed with empty vector, suggesting the protective role of enhanced OsGSTU4 expression. The exposure of plants to various abiotic stresses leads to oxidative damages [1]. Thus, plants employ the components of oxidative defense system to minimize the damaging effect. They have a antioxidant system to balance over-production of ROS and their scavenging to maintain them at required level. Antioxidant enzymes, such as GST, GRX, SOD and CAT etc. catalyze scavenging of ROS and combat damages induced by abiotic stresses. Increased GST activity due to the over-expression of *OsGSTU4* can eliminate/reduce the level of ROS species under stress conditions. Several reports showed enhanced tolerance to various abiotic stresses, such as salinity, oxidative, osmotic and drought, with increased GST activity due to the over-expression of GSTs [31], [32], [59]–[62]. Based on these observations, we can conclude that lower accumulation of H_2_O_2_ and O^2**•–**^ due to the higher antioxidant (GST) activity, at least in part, might directly or indirectly contribute to the enhanced stress tolerance of *OsGSTU4* over-expression transgenic Arabidopsis plants.

In conclusion, we investigated the role of a rice tau class GST gene, *OsGSTU4*, in response to various abiotic stresses. In *E. coli*, the expression of *OsGSTU4* imparts tolerance to various stresses. The transgenic Arabidopsis plants expressing *OsGSTU4* also showed better seed germination, root and plant growth and increased capacity to retain chlorophyll content in plant leaves as compared to WT plants under salinity and oxidative stress conditions. The over-expression Arabidopsis transgenic lines showed lower accumulation of ROS and increased GST activity. The increased salinity and oxidative stress tolerance might be related to the higher level of GST activity in transgenic lines due to the overexpression of *OsGSTU4*. These results suggest that *OsGSTU4* can be used as a suitable candidate to improve salinity and oxidative stress tolerance in crop plants.

## Supporting Information

Figure S1 **Sub-cellular localization of OsGSTU4 in Arabidopsis leaf epidermis.** Images were observed in GFP filter by confocal microscopy after 24 h of incubation.
(PDF)

Figure S2 **Growth/phenotype of** ***OsGSTU4*** **over-expression transgenic and WT plants under normal growth condition.** (A) One-month-old plants grown in plastic pots in vermiculite. (B) Appearance of WT and transgenic plants in mature stage.
(PDF)

Figure S3 **Leaf disc assays showing salinity and oxidative stress tolerance.** Effect of salinity (A) and oxidative stress (B) on the leaves of transgenic and WT plants. For stress treatment at least 20 plants and 4–5 leaves from each line were used.
(PDF)

Figure S4 **Abiotic stress tolerance of** ***OsGSTU4*** **expressing transgenic lines.** Phenotypic characterization of four-week-old transgenic and WT under salinity (A) and oxidative (B) stress. Photographs of plant phenotypes were taken after one week of stress treatment using 200 mM NaCl and 25 μM MV.
(PDF)

Table S1 **List of primers used for real time PCR analysis in this study.**
(PDF)

Table S2 **List of genes differentially expressed in transgenic line as compared to wild-type.**
(PDF)
