# Supplementary material for: Over-Expression of a Rice Tau Class Glutathione S-Transferase Gene Improves Tolerance to Salinity and Oxidative Stresses in Arabidopsis
Source: PLoS One. 2014 Mar 24;9(3):e92900. doi: 10.1371/journal.pone.0092900 (PMC3963979; doi:10.1371/journal.pone.0092900)

Figure S1. Sub-cellular localization of OsGSTU4 in Arabidopsis leaf epidermis. Images were observed in GFP filter by confocal microscopy after 24 h of incubation.

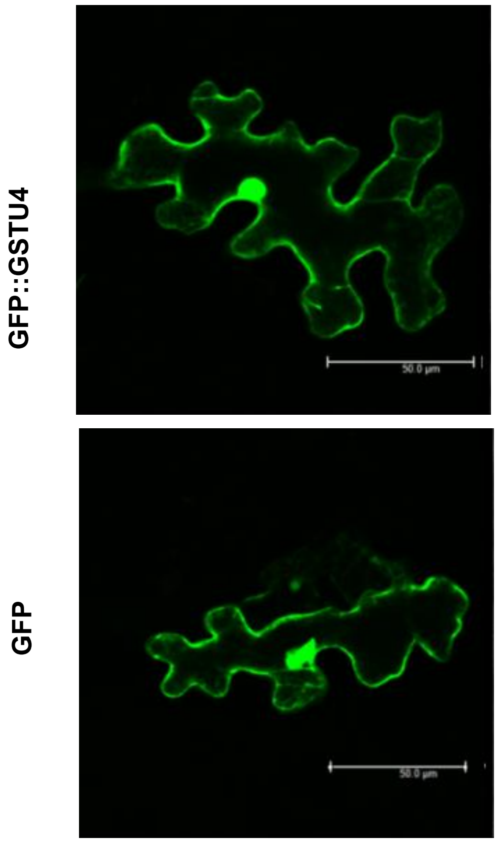

Supplement: Figure S1 — Sub-cellular localization of OsGSTU4 in Arabidopsis leaf epidermis. Images were observed in GFP filter by confocal microscopy after 24 h of incubation. (PDF) [file pone.0092900.s001.pdf]
