# Supplementary material for: Over-Expression of a Rice Tau Class Glutathione S-Transferase Gene Improves Tolerance to Salinity and Oxidative Stresses in Arabidopsis
Source: PLoS One. 2014 Mar 24;9(3):e92900. doi: 10.1371/journal.pone.0092900 (PMC3963979; doi:10.1371/journal.pone.0092900)

Figure S2. Growth/phenotype of *OsGSTU4* over-expression transgenic and WT plants under normal growth condition. (A) One-month-old plants grown in plastic pots in vermiculite. (B) Appearance of WT and transgenic plants in mature stage.

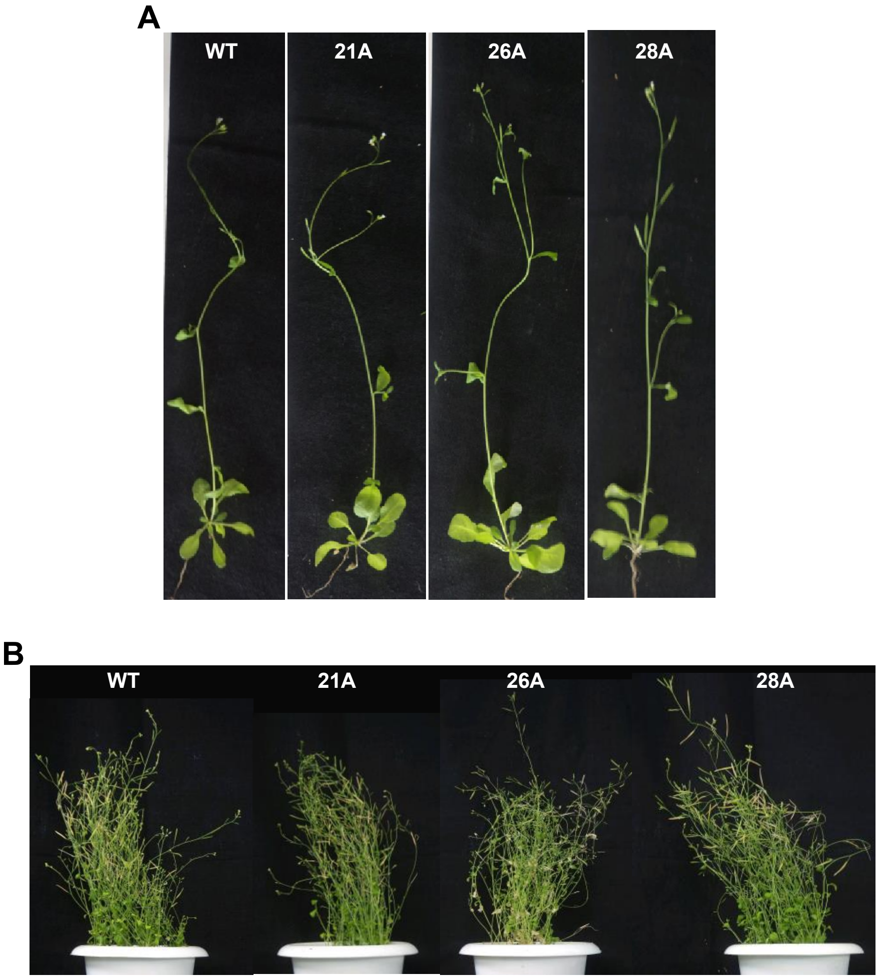

Supplement: Figure S2 — Growth/phenotype of OsGSTU4 over-expression transgenic and WT plants under normal growth condition. (A) One-month-old plants grown in plastic pots in vermiculite. (B) Appearance of WT and transgenic plants in mature stage. (PDF) [file pone.0092900.s002.pdf]
