# Supplementary material for: Over-Expression of a Rice Tau Class Glutathione S-Transferase Gene Improves Tolerance to Salinity and Oxidative Stresses in Arabidopsis
Source: PLoS One. 2014 Mar 24;9(3):e92900. doi: 10.1371/journal.pone.0092900 (PMC3963979; doi:10.1371/journal.pone.0092900)

Figure S3. Leaf disc assays showing salinity and oxidative stress tolerance. Effect of salinity (A) and oxidative stress (B) on the leaves of transgenic and WT plants. For stress treatment at least 20 plants and 4-5 leaves from each line were used.

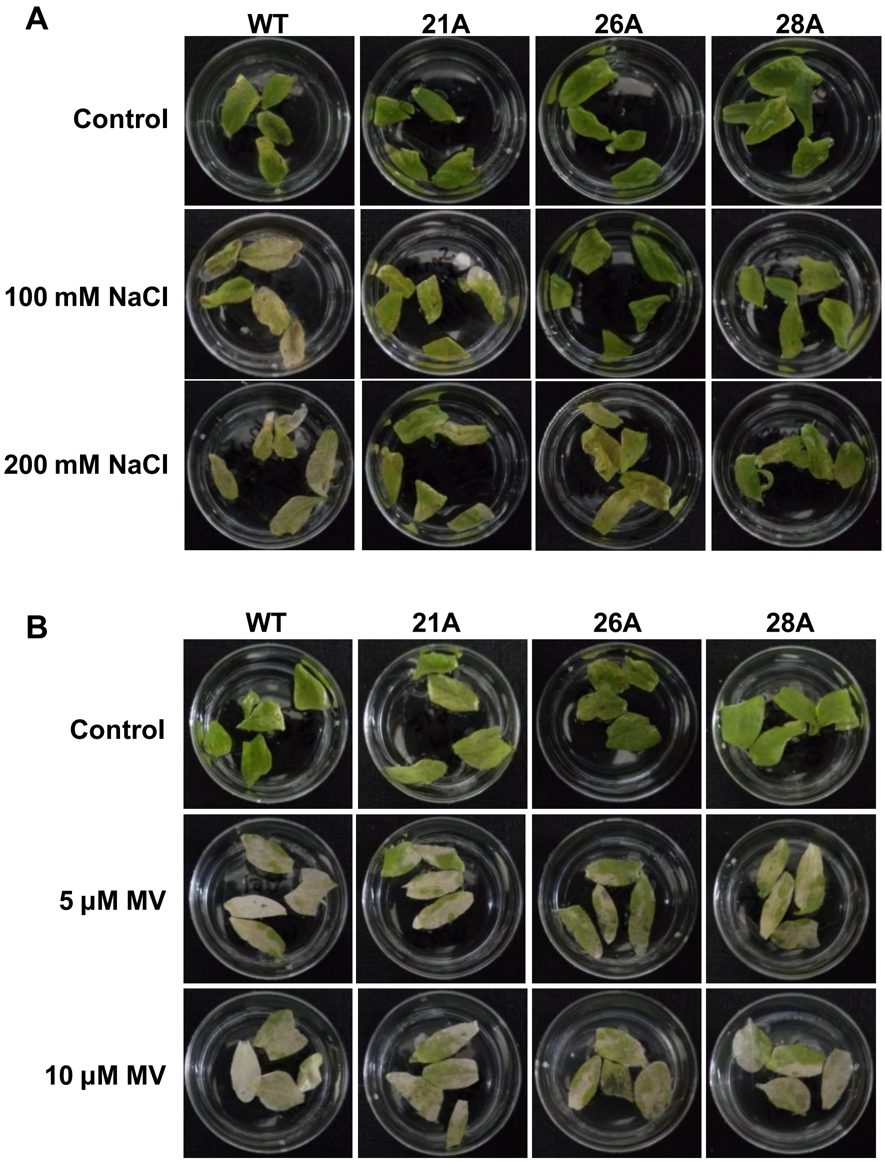

Supplement: Figure S3 — Leaf disc assays showing salinity and oxidative stress tolerance. Effect of salinity (A) and oxidative stress (B) on the leaves of transgenic and WT plants. For stress treatment at least 20 plants and 4–5 leaves from each line were used. (PDF) [file pone.0092900.s003.pdf]
