# Supplementary material for: Over-Expression of a Rice Tau Class Glutathione S-Transferase Gene Improves Tolerance to Salinity and Oxidative Stresses in Arabidopsis
Source: PLoS One. 2014 Mar 24;9(3):e92900. doi: 10.1371/journal.pone.0092900 (PMC3963979; doi:10.1371/journal.pone.0092900)

Figure S4. Abiotic stress tolerance of *OsGSTU4* expressing transgenic lines. Phenotypic characterization of four-week-old transgenic and WT under salinity (A) and oxidative (B) stress. Photographs of plant phenotypes were taken after one week of stress treatment using 200 mM NaCl and 25 εM MV.

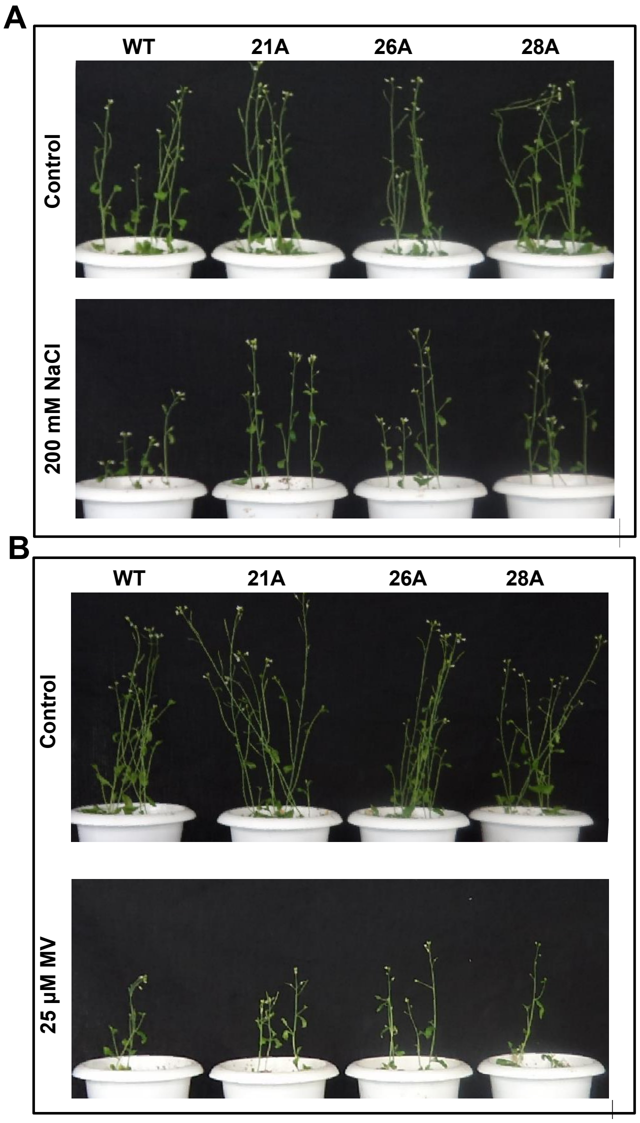

Supplement: Figure S4 — Abiotic stress tolerance of OsGSTU4 expressing transgenic lines. Phenotypic characterization of four-week-old transgenic and WT under salinity (A) and oxidative (B) stress. Photographs of plant phenotypes were taken after one week of stress treatment using 200 mM NaCl and 25 μM MV. (PDF) [file pone.0092900.s004.pdf]
