# Supplementary material for: Over-Expression of a Rice Tau Class Glutathione S-Transferase Gene Improves Tolerance to Salinity and Oxidative Stresses in Arabidopsis
Source: PLoS One. 2014 Mar 24;9(3):e92900. doi: 10.1371/journal.pone.0092900 (PMC3963979; doi:10.1371/journal.pone.0092900)
Supplement: Table S1 — List of primers used for real time PCR analysis in this study. (PDF) [file pone.0092900.s005.pdf]

Table S1. List of primers used for real time PCR analysis in this study.

Gene identifier	Primer sequence
At1g17170	F-TCCACAATGGTAAACCGGTATG
	R-TCGGGCCAAGTCTCGTCTAT
At4g21990	F-GTCAACGGGACAGCCTCAGT
	R-TGCCTGCTCAAGTTCACAACA
At1g69930	F-CATCCTTCCCTCTGATCCATTT
	R-TTCATCGATATAGACGTCCCAAAA
At3g28740	F-AGAATGGGCGATGTCGAATTT
	R-TTTCGGTCTTTGCCTTTCGTA
Os10g38495 (OsGSTU4)	F-CTACGTCGACGACAAGTTCGTT
	R-TCCTCCGTCTTGCCTCTGAA
PP2A	F-TAACGTGGCCAAAATGATGC
	R-GTTCTCCACAACCGCTTGGT
UBQ5	F-ACCACTTCGACCGCCACTACT
	R-ACGCCTAAGCCTGCTGGTT
