# Supplementary material for: Over-Expression of a Rice Tau Class Glutathione S-Transferase Gene Improves Tolerance to Salinity and Oxidative Stresses in Arabidopsis
Source: PLoS One. 2014 Mar 24;9(3):e92900. doi: 10.1371/journal.pone.0092900 (PMC3963979; doi:10.1371/journal.pone.0092900)
Supplement: Table S2 — List of genes differentially expressed in transgenic line as compared to wild-type. (PDF) [file pone.0092900.s006.pdf]

Table S2. List of genes differentially expressed in transgenic line as compared to wild-type.

Probe set ID	Gene identifier	Gene description	p - value	Fold change	Regulation
267053_s_at	AT5G02780	In2-1 protein, putative	0.01	2.2396953	up
266967_at	AT1G65690	harpin-induced protein-related / HIN1-related / harpin-responsive protein-related	0.01	2.2465532	up
266290_at	AT3G02800	phosphoprotein phosphatase	0.01	2.566293	up
266294_at	AT5G20790	similar to unknown protein [Arabidopsis thaliana] (TAIR:AT3G43110.1)	0.01	2.2048268	up
266296_at	AT2G15490	UGT73B4; UDP-glycosyltransferase/ transferase, transferring glycosyl groups	0.01	2.0380855	up
265501_at	AT1G26380	FAD-binding domain-containing protein	0.01	2.8756747	up
265214_at	AT3G13950	similar to Ankyrin [Medicago truncatula] (GB:ABN08906.1)	0.01	2.1825197	up
263210_at	AT1G17170	ATGSTU24 (ARABIDOPSIS THALIANA GLUTATHIONE S-TRANSFERASE (CLASS TAU) 24)	0.04	2.137459	up
263184_at	AT4G21990	APR3 (APS REDUCTASE 3)	0.02	2.0007417	up
263073_at	AT2G39530	integral membrane protein, putative	0.01	2.1286125	up
262930_at	AT2G17500	auxin efflux carrier family protein	0.02	2.129894	up
262903_at	AT5G45630	similar to unknown protein [Arabidopsis thaliana] (TAIR:AT4G18980.1)	0.02	2.102777	up
262911_s_at	AT2G29500	17.6 kDa class I small heat shock protein (HSP17.6B-CI)	0.01	2.3026447	up
262518_at	AT5G61160	AACT1 (ANTHOCYANIN 5-AROMATIC ACYLTRANSFERASE 1); transferase	0.02	3.00391	up
262229_at	AT3G17790	ATACP5 (acid phosphatase 5); acid phosphatase/ protein serine/threonine phosphatase	0.03	2.2053978	up
262047_at	AT4G38080	hydroxyproline-rich glycoprotein family protein	0.01	2.3538074	up
261934_at	AT2G45220	pectinesterase family protein	0.01	2.0003695	up
261562_at	AT3G49845	contains InterPro domain XYPPX repeat (InterPro:IPR006031)	0.01	2.0535083	up
261021_at	AT1G05000	tyrosine specific protein phosphatase family protein	0.03	2.6180785	up
260405_at	AT1G59860	[AT1G59860, 17.6 kDa class I heat shock protein (HSP17.6A-CI)]	0.01	2.1953585	up
258586_s_at	AT4G12500	protease inhibitor/seed storage/lipid transfer protein (LTP) family protein	0.01	2.342929	up
258277_at	AT3G04320	AT3G04320, endopeptidase inhibitor	0.02	2.6018543	up
258203_at	AT2G38380	[AT2G38380, peroxidase 22 (PER22) (P22) (PRXEA)	0.03	2.5016282	up
258158_at	AT5G13930	ATCHS/CHS/TT4 (CHALCONE SYNTHASE); naringenin-chalcone synthase	0.01	2.0128002	up
258133_at	AT1G80160	lactoylglutathione lyase family protein / glyoxalase I family protein	0.01	2.6152046	up
256839_at	AT2G29420	ATGSTU7 (GLUTATHIONE S-TRANSFERASE 25); glutathione transferase	0.02	2.5213528	up
256589_at	AT3G28740	cytochrome P450 family protein	0.01	2.073456	up
255284_at	AT3G24500	ATMBF1C/MBF1C (MULTIPROTEIN BRIDGING FACTOR 1C)	0.03	2.194467	up
254819_at	AT3G60420	similar to unknown protein [Arabidopsis thaliana] (TAIR:AT3G60450.1)	0.01	2.2578611	up
254343_at	AT2G29490	ATGSTU1 (GLUTATHIONE S-TRANSFERASE 19); glutathione transferase	0.01	2.2355654	up

253268_s_at	AT1G05560	UGT1 (UDP-glucosyl transferase 75B1)	0.01	2.3629797	up
253024_at	AT1G22400	ATUGT85A1/UGT85A1 (UDP-GLUCOSYL TRANSFERASE 85A1)	0.02	2.071739	up
252984_at	AT3G26830	PAD3 (PHYTOALEXIN DEFICIENT 3); oxygen binding	0.01	2.9650905	up
252320_at	AT1G69930	ATGSTU11 (Arabidopsis thaliana Glutathione S-transferase (class tau) 11)	0.02	2.0983686	up
252222_at	AT4G37990	ELI3-2 (ELICITOR-ACTIVATED GENE 3)	0.01	2.008402	up
251400_at	AT4G04610	APR1 (PAPS REDUCTASE HOMOLOG 19)	0.01	2.8856394	up
250983_at	AT4G34131	[AT4G34131, UGT73B3 (UDP-GLUCOSYL TRANSFERASE 73B3)	0.03	3.1259625	up
250351_at	AT1G10585	transcription factor	0.01	2.117144	up
250207_at	AT1G68620	hydrolase	0.04	2.0338898	up
248959_at	AT1G01750	actin-depolymerizing factor, putative	0.01	2.2465825	up
247573_at	AT5G12030	AT-HSP17.6A (Arabidopsis thaliana heat shock protein 17.6A)	0.02	2.1289144	up
246001_at	AT3G22930	calmodulin, putative	0.03	2.390505	up
245148_at	AT1G59950	aldo/keto reductase, putative	0.01	2.0409153	up
257517_at	AT3G48580	xyloglucan:xyloglucosyl transferase, putative	0.02	2.4532588	up
257536_at	AT3G16330	similar to unknown protein [Arabidopsis thaliana] (TAIR:AT1G52140.1)	0.02	2.0232038	up
260118_s_at	ATCG00560	PSII L protein	0.02	-2.071837	down
259839_at	ATCG00870	[ATCG00870, hypothetical protein];[ATCG01270, hypothetical protein]	0.05	-2.0886283	down
259391_s_at	AT2G23170	GH3.3; indole-3-acetic acid amido synthetase	0.01	-2.3084345	down
258218_at	ATCG00490	large subunit of RUBISCO.	0.02	-2.3944995	down
248812_at	AT5G47330	palmitoyl protein thioesterase family protein	0.02	-2.258506	down
248282_at	AT1G52190	proton-dependent oligopeptide transport (POT) family protein	0.01	-2.0306478	down
247474_at	AT5G62280	similar to unknown protein [Arabidopsis thaliana] (TAIR:AT2G45360.1)	0.01	-2.1890955	down
245076_at	AT5G52900	similar to unnamed protein product [Vitis vinifera] (GB:CAO49548.1)	0.01	-2.1388087	down
245002_at	ATCG00300	encodes PsbZ, which is a subunit of photosystem II	0.02	-2.6225924	down
245003_at	ATCG00280	chloroplast gene encoding a CP43 subunit of the photosystem II reaction center	0.05	-5.4850087	down
245004_at	ATMG00080	ATMG00080, encodes a mitochondrial ribosomal protein L16	0.01	-4.54835	down
245015_at	AT3G17998	[AT3G17998, CPuORF30 (Conserved peptide upstream open reading frame 30)]	0.02	-2.5040536	down
245022_at	AT5G18700	[AT5G18700, EMB3013 (EMBRYO DEFECTIVE 3013); kinase]	0.02	-2.1528046	down
244969_at	AT1G06360	[AT1G06360, fatty acid desaturase family protein]	0.02	-2.280616	down
244990_s_at	ATCG00270	PSII D2 protein	0.03	-4.179902	down
244936_at	ATCG00650	chloroplast-encoded ribosomal protein S18	0.03	-2.0119448	down
244938_at	ATCG01120	encodes a chloroplast ribosomal protein S15	0.03	-2.0435781	down
244944_s_at	ATCG01100	NADH dehydrogenase ND1	0.01	-2.320282	down
